# Short-term exposure to ozone and asthma exacerbation in adults: A longitudinal study in China

**DOI:** 10.3389/fpubh.2022.1070231

**Published:** 2023-01-06

**Authors:** Xinyi Fang, Suijie Huang, Yixiang Zhu, Jian Lei, Yanyi Xu, Yue Niu, Renjie Chen

**Affiliations:** ^1^Key Laboratory of Public Health Safety of the Ministry of Education, NHC Key Laboratory of Health Technology Assessment, School of Public Health, Fudan University, Shanghai, China; ^2^Guangzhou Homesun Medical Technology Co. Ltd., Guangzhou, Guangdong Province, China

**Keywords:** ozone, asthma exacerbation, lung function, diurnal peak expiratory flow variation, longitudinal study

## Abstract

**Background:**

The relationships between short-term ozone exposure and the acute exacerbations of asthma in adults have not been fully studied. Existing studies commonly ignored the effects of ozone on mild or early asthma exacerbations.

**Objective:**

To investigate the associations between short-term ozone exposure and asthma exacerbations in Chinese adults.

**Methods:**

We administered health management for adult asthma patients through the Respiratory Home Platform and required them to monitor their lung function every morning and evening by themselves. Finally, a total of 4,467 patients in 18 Chinese cities were included in the current analyses, with 79,217 pairs of lung function records. The maximum daily 8-h average ozone concentrations were collected from fixed-site air quality monitoring stations. We calculated diurnal peak expiratory flow (PEF) variation using morning and evening measurements of PEF and then defined different severity of asthma exacerbations with diurnal PEF variations >10, 15, and 20%, respectively. A binomial distributed generalized additive mixture model combined with distribution non-linear models was applied to examine the association of ozone with asthma exacerbations. We further conducted stratified analyses by sex, age, season of lung function tests, and region.

**Measurements and results:**

We found that short-term ozone exposure was independently associated with an elevated risk of asthma exacerbations defined by lung function and the effects could last for about 2 days. At lag 0–2 days, each 10 μg/m^3^ increment in ozone concentration was associated with odds ratios of 1.010 [95% confidence interval (CI): 1.003, 1.017], 1.014 (95% CI: 1.005, 1.023), and 1.017 (95% CI: 1.006, 1.028) for asthma exacerbations that were defined by diurnal PEF variation over 10, 15, and 20%, respectively. The associations remained significant after adjusting for other pollutants, and became unstable when using 24-h average ozone concentration. We also found that the associations were relatively stronger in males, those aged 45 years and older, and in the warm season.

**Conclusions:**

Our results suggest that short-term ozone exposure can increase the risk of asthma exacerbations, even in the early stage of exacerbation. Male and older asthma patients may be more vulnerable to ozone air pollution, especially in the warm season.

## 1. Introduction

By 2019, ~262 million people worldwide suffered from asthma, one of the most common respiratory diseases, and nearly half a million died from asthma each year (1). In China, the prevalence of asthma in people aged ≥ 20 years was 4.2% through 2019, with a total of 45.7 million asthmatic patients (2). Asthma tends to progress over time and tends to present with recurrent relapses and remissions. At present, the overall goal of asthma management is to get asthma under control, that is, to minimize the symptom burden and the risks of acute exacerbations (3, 4). Research suggested that daily monitoring of lung function and restricting exposure to risk factors are essential for asthma health management (5, 6). Unlike childhood asthma, adult-onset asthma exhibits poorer control and a rapid decline in lung function. Therefore, recognizing risk factors for adult asthma exacerbations will aid in the prevention and management of acute asthma attack in adults.

As a highly oxidizing and reactive gas, inhaled ozone is considered to be one of toxicants for the respiratory tract, which has been linked to a variety of adverse respiratory outcomes, such as a higher risk of respiratory mortality, increased incidence of respiratory diseases, and exacerbation of respiratory irritations (7, 8). Previous epidemiological studies have investigated the associations between ozone pollution and acute asthma exacerbations (9–11). For example, a national study in US and multi-city studies in Texas have associated short-term ozone concentration with asthma hospitalization (10, 12, 13). Associations of ozone with asthma emergency room visits were also reported in many studies (14, 15). A recent meta-analysis concluded that pooled relative risks per 10 μg/m^3^ in ozone concentration were 1.014 for asthma hospitalization and 1.006 for asthma emergency room visits (9). Moreover, Pepper et al. also found that ambient ozone concentration was positively associated with the use of short-acting beta-2 agonists, a medication for asthma attacks, providing additional evidence for ozone exposure and asthma exacerbations (11).

Collectively, almost all these studies used emergency room visits and hospital admissions to indicate asthma exacerbation, which may limit capacity to capture acute exacerbations without noticeable symptoms. As a global authority on the prevention and treatment of asthma, the Global Initiative for Asthma (GINA) believes that lung function measures, such as peak expiratory flow (PEF), are more responsive and reliable than symptoms (e.g., coughing or shortness of breath) in reflecting asthma exacerbation during the acute phase (5). In addition, most previous studies focused on childhood asthma and most have been carried out in Europe and the United States. The relationships between short-term ozone exposure and the acute exacerbations of asthma in adults have not been adequately studied, especially in China.

Therefore, we conducted this multi-city longitudinal study with repeated measurements to investigate the possible associations of short-term ozone exposure with asthma exacerbations defined by individual lung function in Chinese adults. We further assessed whether these associations were modified by sex, age, the season when lung function was tested, and the region where the participants lived.

## 2. Materials and methods

### 2.1. Study design and population

This is a dynamic longitudinal study with repeated measurements in adults with asthma from the Respiratory Home Platform. The entire study began on January 1, 2017 and ended on December 31, 2020. A total of 11,599 physician-diagnosed mildly asthmatic patients registered on this platform and received daily follow-ups for asthma management. The median duration of follow-ups was 31 days. All patients were instructed to use a portable spirometer to consecutively monitor their lung function twice daily. Baseline information on individual characteristics [including sex, age, body mass index (BMI)] and residential address was also obtained through the platform. As described previously (16, 17), we excluded those with unknown baseline information, those younger than 18 years or older than 90 years, and those living outside urban areas. The Institutional Review Board in School of Public Health, Fudan University, reviewed and approved the study protocol (IRB#2021-04-0889). Informed consent was obtained from all participants.

### 2.2. Health outcomes

Each registered patient was assigned a portable and smart spirometer (BreathHome Inc., China) for daily monitoring of their lung function. These devices meet the requirements of American Thoracic Society and European Respiratory Society (ATS/ERS). After receiving technical training from their physicians, all participants were able to use the spirometer correctly to measure their lung function by themselves in strict accordance with the standard procedures (18). Participants were required to complete a lung function test in the morning (approximately between 6 and 10 a.m.) and again in the evening (approximately between 6 and 10 p.m.) daily at home. For each round of test, participants were asked to conduct three consecutive measurements of lung function, and the measurements would be recorded and uploaded to the Breath Home platform. If a patient did not complete two rounds of lung function tests in 1 day, the records on that day for this patient would be excluded from the current analysis. We also excluded participants who had <3 days of lung function monitoring.

In the present study, we downloaded data on PEF from the Respiratory Home Platform and calculated the diurnal PEF variation according to the following equation (16):


(1) $$\begin{array}{l} diurnal\;PEF\;variation=\frac{\left|PEF_{m}-PEF_{e}\right|}{0.5\times \left(PEF_{m}+PEF_{e}\right)}\times 100\% \end{array}$$

where PEF_m_ and PEF_e_ refer to the highest PEF value measured in the morning and the evening on the same day, respectively.

We then defined asthma exacerbation based on the calculated diurnal PEF variation. In many previous studies, diurnal PEF variation has been used to indicate the severity of asthma (16, 19). The GINA guidelines and previous studies showed that diurnal PEF variation exceeding 10, 15, and 20% demonstrated reversible airflow restriction, airway inflammation, and severe asthma attack, respectively (5, 16, 20). Therefore, we used these cut-off values to compute three binary variables to indicate acute exacerbation of asthma in this study. The broad definition (cut-off value = 10%) can simultaneously capture mild, moderate, and relatively severe asthma exacerbation, whereas the strict definition (cut-off value = 20%) limits outcomes of interest to relatively severe exacerbations.

### 2.3. Environmental measurements

Ambient ozone concentrations were measured by the state-controlled air quality monitoring stations in real-time and were uploaded on the China's National Urban Air Quality Real-time Publishing Platform. All monitoring stations are located far away from buildings, factories, and major traffic roads, and thus are not affected by local pollution sources and can truly reflect ambient ozone levels in the city. We obtained hourly ozone data from this platform and assigned data from the monitoring station nearest to the participants' residential address (median distance of 3.3 km) to the corresponding participants. Given that the diurnal PEF variation reflected the variability of lung function throughout the day, we used the maximum daily 8-h average concentration as a daily exposure metric for ozone, which was widely used in epidemiological studies (21, 22). We calculated the maximum daily 8-h average concentration only when the hourly measurements covered more than 75% of this time period. In addition, we calculated the 24-h average ozone concentration for sensitivity analysis.

We also obtained other air pollutants data from the platform, including particulate matter with an aerodynamic diameter <2.5 μm (PM_2.5_), nitrogen dioxide (NO_2_), sulfur dioxide (SO_2_), and carbon monoxide (CO). We calculated 24-h average concentrations for these pollutants. Meteorological data were obtained from the China Meteorological Data Sharing Service, including 24-h average temperature and relative humidity. In statistical analyses, we excluded outliers of the meteorological data and pollutant data when they are greater than (mean + 3 × standard deviation) or less than (mean – 3 × standard deviation).

### 2.4. Statistical analyses

#### 2.4.1. Main analyses

Generalized additive mixed (GAM) models with binomial distribution were employed to estimate the associations of ozone exposure with asthma exacerbation. In the main analysis, we introduced three dichotomous variables of asthma exacerbation defined by diurnal PEF variation as a dependent variable, separately. Participants' ID number was included as a random effect intercept. We fitted a distributed non-linear model (DLNM) to generate a cross-basis matrix of ozone, and then incorporated it in the GAM models as an independent variable. The DLNM has an advantage of exploring the associations between ozone and asthma exacerbation along two dimensions of exposure variation and lag, simultaneously. Specifically, we used a linear function to characterize the exposure-response dimension, since most previous studies have demonstrated the linearity of the health effects of ozone (22–25), and used a B-spline function with 2 degrees of freedom to characterize the lag-response dimension. A maximum lag of 7 days was used in the DLNM to capture the potential lagged effects of ozone. In addition, other covariates were also included: (1) individual characteristics (sex, age and BMI); (2) season of lung function tests; (3) regions (southern and northern China); (4) a natural spline function for calendar days from the start of the study to the end of the study with 4 degrees of freedom per year; and (5) natural splines of 8-d moving average temperature and relative humidity over lag 0–7 days, both with 3 degrees of freedom. Based on the lag pattern, the lag period with the most prominent cumulative effect would be selected as the main lag period and used in the main model and subsequent analyses.

#### 2.4.2. Sensitivity analyses

We performed three sensitivity analyses to test the robustness of our results. First, as in previous environmental epidemiological studies, we fitted two-pollutant models to additionally control for possible confounding by PM_2.5_, NO_2_, SO_2_, and CO. Second, we controlled for temperature and relative humidity using alternative lag periods, including the average values on the present day of lung function tests (lag 0 day) and moving averages from the present day to the previous 3 days (lag 0–3 days). Third, to examine whether our findings were affected by the choice of exposure metrics, we replaced the maximum daily 8-h average ozone concentration with the 24-h average concentration in the main models.

#### 2.4.3. Additional analyses

Then, we performed subgroup analyses by sex (male and female), age (18–44 and 45–88 years), season of lung function tests (warm and cool), and region (southern China or northern China) to examine their potential effect modifications. We then assessed the statistical significance of between-group differences in the associations of ozone with asthma exacerbation by calculating their 95% confidence intervals (CIs), as in previous studies (17, 26). In addition, we examined the associations between the maximum daily 8-h average ozone concentration and diurnal PEF variation using linear mixed-effect models.

Last, we plotted the exposure-response curves between ozone and asthma exacerbation. To fully capture the possible non-linear associations, we used natural cubic splines with 2 degrees of freedom for both lag-response dimension and exposure-response dimension in the DLNM. Other parameters in the main model remained unchanged.

All statistical analyses were conducted in R software (Version 4.3.1). All tests were two-sided and *p* < 0.05 were regarded statistically significant. The results were shown as odds ratios (ORs) of asthma exacerbation and their 95% CIs in relation to a 10 μg/m^3^ increase in ambient ozone concentration.

## 3. Results

### 3.1. Descriptive statistics

**Figure 1:**
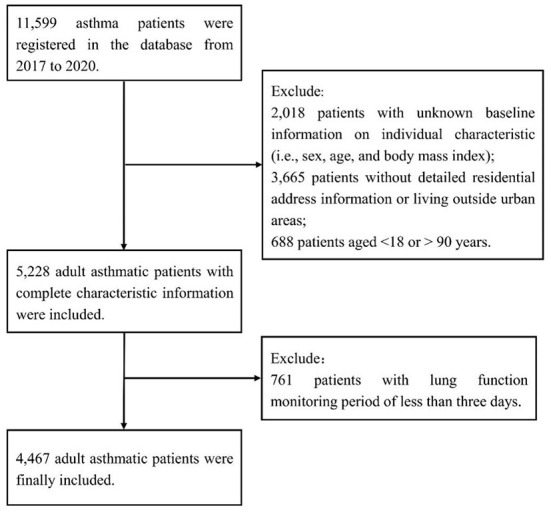
Flow chart of the inclusion and exclusion of adult asthma patients.

As described previously (16, 17), we excluded 2,018 patients with unknown information on individual characteristics, 3,665 patients without detailed residential address information or living outside urban areas, and 688 patients aged < 18 or >90 years, and we further excluded 761 patients with lung function monitoring period of < 3 days (Figure 1). The remaining 4,467 patients were included in the present study and were distributed across 18 Chinese cities (Supplementary Figure 1). A total of 79,217 pairs (morning and evening) of lung function records were included, with a mean number of 18 pairs of records per person. The mean age of the study population was 44.2 years (standard deviation, 13.8 years.) and the mean BMI was 23.0 kg/m^2^ (standard deviation, 3.5 kg/m^2^). Of the 4,467 patients, 59.7% were female. A total of 24.4, 13.6, and 8.2% of lung function records met the broad, moderate, and strict definitions of asthma exacerbation in this study, i.e., diurnal PEF variation of more than 10, 15 and 20%, respectively. The proportions of records with diurnal PEF variation >10, 15 and 20% were similar between males and females. The proportions of the three indicators were larger in the people aged between 45 and 88 years than in the people aged between 18 and 44 years (Table 1).

**Table 1:** Summary of diurnal PEF variations, stratified by sex and age.

**Variables**	**Total**	**Sex**		**Age**	

		**Male**	**Female**	**45-88**	**18-44**
*N*	79,217	31,710	47,507	42,165	37,052
Diurnal PEF variation > 10%, *N* (%)	19,313 (24.4%)	7,737 (24.4%)	11,498 (24.2%)	11,379 (27.0%)	7,856 (21.2%)
Diurnal PEF variation > 15%, *N* (%)	10,742 (13.6%)	4,321 (13.6%)	6,417 (13.5%)	6,493 (15.4%)	4,245 (11.5%)
Diurnal PEF variation >20%, *N* (%)	6,511 (8.2%)	2,592 (8.2%)	3,919 (8.2%)	4,013 (9.5%)	2,498 (6.7%)

N, number of paired lung function records; PEF, peak expiratory flow.

Table 2 summarizes the descriptive results of the environmental data. The ambient concentration of ozone ranged from 4.0 to 264.5 μg/m^3^ during the study period, with a mean concentration of 89.4 μg/m^3^ (standard deviation, 55.6 μg/m^3^). The concentration of ozone was higher in the warm season than in the cool season, and higher in the southern China than in the northern China (Supplementary Table 1). During the same period, the average concentrations of PM_2.5_, NO_2_, SO_2_ and CO were 46.6 μg/m^3^, 44.8 μg/m^3^, 9.1 μg/m^3^ and 0.9 mg/m^3^, respectively. Ambient ozone concentration was weakly associated with other air pollutants and relative humidity while moderately and positively associated with temperature (Supplementary Table 2).

**Table 2:** Descriptive statistics of air pollutants and meteorological conditions in 18 Chinese cities, 2017–2020.

**Variables**	**Mean**	**SD**	**Min**	**P_25_**	**Median**	**P_75_**	**Max**
**Air pollutants**							
O_3_, μg/m^3^	89.4	55.6	4.0	49.3	76.8	121.3	264.5
PM_2.5_, μg/m^3^	46.6	37.1	3.8	21.0	36.3	59.8	246.4
NO_2_, μg/m^3^	44.8	20.9	7.0	29.0	41.0	56.4	127.9
SO_2_, μg/m^3^	9.1	8.2	1.1	3.5	6.7	11.7	61.3
CO, mg/m^3^	0.9	0.4	0.2	0.6	0.8	1.1	3.0
**Meteorologic conditions**							
Temperature, °C	14.6	11.5	−13.5	3.8	16.8	25.0	32.3
Relative humidity, %	57.5	22.0	13.0	38.0	59.0	76.0	98.0

SD, standard deviation; Min, minimum; Max, maximum; P_25_, the 25^th^ percentile; P_75_, the 75^th^ percentile; O_3_, ozone; PM_2.5_, particulate matter with an aerodynamic diameter < 2.5 μm; SO_2_, sulfur dioxide; NO_2_, nitrogen dioxide; CO, carbon monoxide.

### 3.2. Regression results

**Figure 2:**
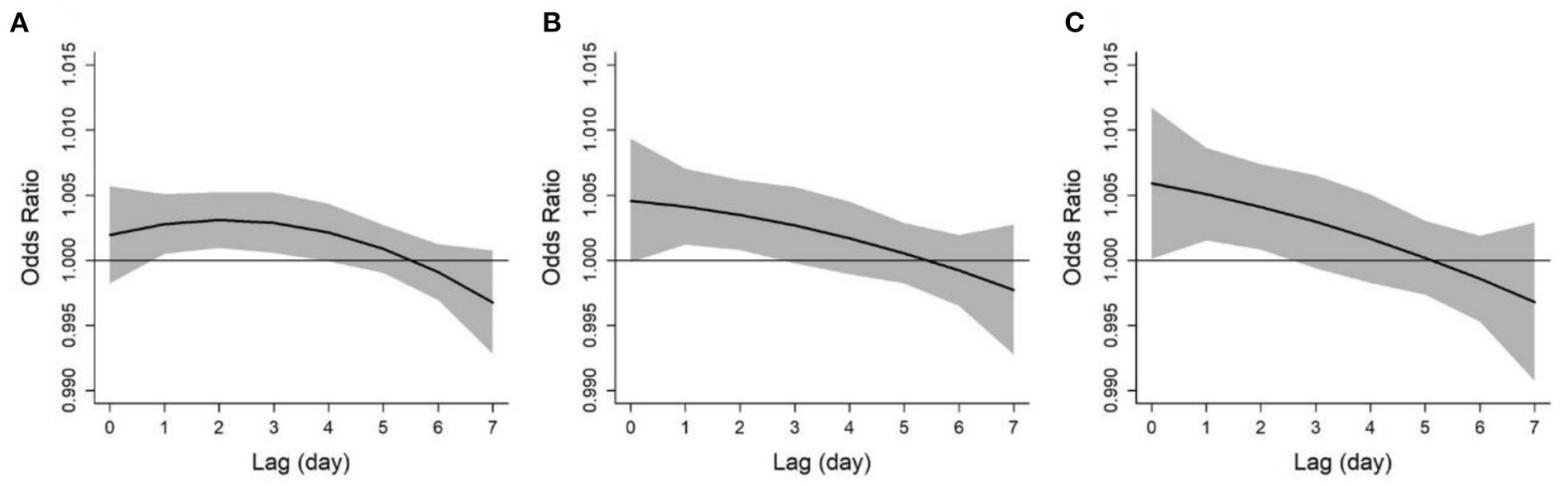
Lag patterns for the associations between ozone and asthma exacerbations defined by diurnal peak expiratory flow variation over 10% **(A)**, 15% **(B)**, and 20% **(C)**. The solid lines and shaded areas represent odds ratios and the 95% confidence intervals, respectively.

Figure 2 shows the lag structure of the effects of ozone on asthma exacerbation over a lag period of 0–7 days. The lag-response curves were similar for asthma exacerbation in different definitions, all showing a linear downward trend. Specifically, the effect of ozone on asthma exacerbation appeared on the current day of exposure, lasted for about 2 days, then decreased in magnitude and became insignificant. Therefore, we selected lag 0–2 days as the main lag period and used it for subsequent analyses.

**Figure 3:**
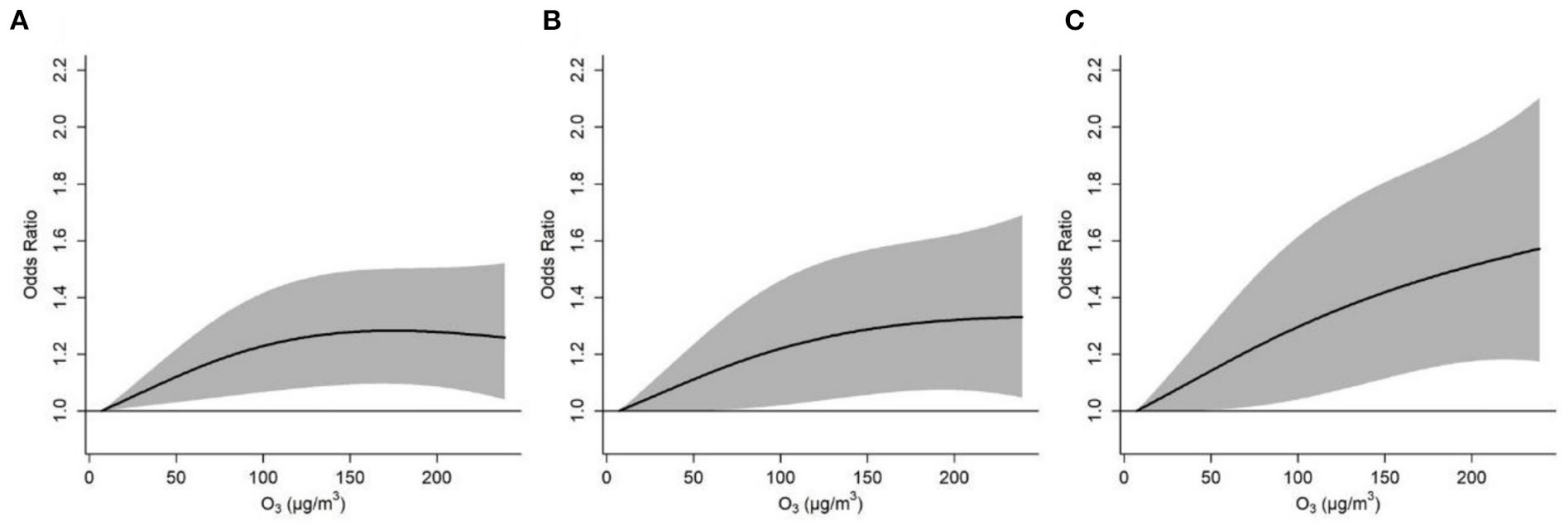
Cumulative exposure-response curves for the associations between ozone concentration and asthma exacerbations defined by diurnal peak expiratory flow variation over 10% **(A)**, 15% **(B)**, and 20% **(C)**, over lag 0–2 days. The solid lines and shaded areas represent odds ratios and the 95% confidence intervals, respectively.

Figure 3 depicts the cumulative exposure-response curves between ozone exposure and acute asthma exacerbation over a lag period of 0–2 days. For asthma exacerbations that were defined by diurnal PEF variation >10 and 15%, respectively, the effects first increased with increasing ozone concentration and then plateaued at higher ozone concentration. However, for severe asthma exacerbation that was defined by diurnal PEF variation over 20%, the effect continued to rise in the whole range of ozone concentration. We observed that each 10 μg/m^3^ increase in ozone concentration over a lag period of 0–2 days was associated with ORs of 1.010 (95%CI: 1.003, 1.017), 1.014 (95%CI: 1.005, 1.023), and 1.017 (95%CI: 1.006, 1.028) for asthma exacerbations that were defined by diurnal PEF variation over 10, 15, and 20%, respectively (Table 3). We also observed a significant association between ozone and diurnal PEF variation, in which diurnal PEF variation increased by 0.04% (95% CI: 0.02%, 0.06%) for every 10 μg/m^3^ increase in ozone concentration (Supplementary Table 3).

**Table 3:** Odds ratios of asthma exacerbations associated with a 10 μg/m^3^ increase in ozone concentration (lag 0–2 days) in the main model and sensitivity analysis.

**Model**	**Diurnal PEF variation > 10%**	**Diurnal PEF variation > 15%**	**Diurnal PEF variation >20%**
Main model	1.010 (1.003, 1.017)	1.014 (1.005, 1.023)	1.017 (1.006, 1.028)
**Two-pollutant models**			
O_3_+PM_2.5_	1.009 (1.002, 1.017)	1.014 (1.005, 1.024)	1.016 (1.005, 1.027)
O_3_+NO_2_	1.010 (1.003, 1.017)	1.013 (1.004, 1.022)	1.017 (1.006, 1.028)
O_3_+SO_2_	1.010 (1.003, 1.017)	1.013 (1.004, 1.022)	1.016 (1.005, 1.027)
O_3_+CO	1.009 (1.002, 1.016)	1.013 (1.004, 1.022)	1.017 (1.006, 1.028)
**Lag periods adjustments** ^a^			
0 d	1.005 (0.999, 1.012)	1.008 (0.999, 1.016)	1.010 (0.999, 1.020)
0–3 d	1.009 (1.002, 1.014)	1.012 (1.004, 1.021)	1.015 (1.004, 1.025)
**Alternative exposure metric**			
24-h average ozone	1.008 (0.999, 1.018)	1.014 (1.002, 1.027)	1.019 (1.003, 1.034)

PEF, peak expiratory flow; O_3_, ozone; PM_2.5_, particulate matter with an aerodynamic diameter < 2.5 μm; SO_2_, sulfur dioxide; NO_2_, nitrogen dioxide; CO, carbon monoxide.

a Adjustments for temperature and relative humidity with different lag periods.

In our sensitivity analyses, we found that the associations between ozone exposure and asthma exacerbation remained almost unchanged in magnitude and remained statistically significant after adjustment for PM_2.5_, SO_2_, NO_2_, and CO (Table 3). Similarly, the effects of ozone maintained statistical significance after using the period of lag 0–3 days for temperature and relative humidity, although the effects were attenuated when using a lag period of 0 days (Table 3). We also found that the effects of 24-h average ozone concentration on asthma exacerbation were comparable in magnitude to the effects of maximum daily 8-h average concentration. However, the association with asthma exacerbation defined by diurnal PEF variation over 10% lost statistical significance when using 24-h average ozone concentration (Table 3).

Subgroup analyses showed that the associations between ozone and asthma exacerbation were statistically significant in males but not in females, in those aged ≥ 45 years but not in those aged < 45 years, and in the warm season but not in the cool season. We observed stronger associations in males, those aged ≥ 45 years, and in the warm season, although all of the differences between subgroups did not reach statistical significance. The results of regional stratification showed no significant differences in associations of ozone with asthma exacerbation in southern and northern China (Table 4). Consistently, we also observed significant and relatively larger effects of ozone on diurnal PEF variation in males, those aged ≥ 45 years, and in the warm season (Supplementary Table 3).

**Table 4:** Odds ratios of asthma exacerbations associated with a 10 μg/m^3^ increase in ozone concentrations, classified by sex, age, season of lung function tests, and region.

	**Number of records**	**Diurnal PEF variation > 10%**	**Diurnal PEF variation > 15%**	**Diurnal PEF variation >20%**
**Sex**				
Male	31,710	1.015 (1.004, 1.026)	1.029 (1.014, 1.043)	1.040 (1.022, 1.058)
Female	47,507	1.005 (0.996, 1.014)	1.001 (0.989, 1.013)	0.999 (0.985, 1.013)
**Age**				
45–88	42,165	1.011 (1.002, 1.021)	1.019 (1.007, 1.030)	1.022 (1.008, 1.036)
18–44	37,052	1.006 (0.995, 1.017)	1.005 (0.991, 1.019)	1.008 (0.991, 1.026)
**Season** ^a^				
Warm	38,197	1.010 (1.001, 1.019)	1.020 (1.008, 1.032)	1.028 (1.013, 1.043)
Cool	41,020	1.006 (0.995, 1.017)	1.001 (0.986, 1.015)	0.995 (0.977, 1.013)
**Region**				
South	21,274	1.015 (1.003, 1.027)	1.014 (0.999, 1.029)	1.019 (1.000, 1.037)
North	57,943	1.007 (0.998, 1.016)	1.014 (1.002, 1.025)	1.015 (1.001, 1.029)

PEF, peak expiratory flow.

a Warm season was defined as May to October; cool season was defined as November to April.

## 4. Discussion

The current longitudinal study included more than 4,000 adult asthma patients in 18 Chinese cities, with nearly 80,000 pairs of lung function records measured in the morning and evening. With this dataset, we found that short-term exposure to ozone was independently associated with an increased risk of asthma exacerbation defined by lung function and the effects of short-term ozone exposure could last for about 2 days. We also found that the associations between ozone exposure and asthma exacerbation were slightly stronger in males, those aged 45 years and older, and in the warm season.

Our main findings were consistent with numbers of previous studies that suggested an association between ozone and asthma exacerbation. In a meta-analysis, Li et al. found that for every 10 μg/m^3^ increment in maximum daily 8-h average ozone concentration, the risk of adult asthma exacerbation rose by 11% (27). The risk of asthma exacerbation attenuated to the null when 24-h average ozone concentration was used (28). In the present study, we also found the maximum daily 8-h average ozone concentration had a more robust association with asthma exacerbation compared with the 24-h average concentration, supporting the use of this metric in the Ambient Air Quality Standard (GB 3095-2012). In addition, we observed that the ozone-asthma associations became larger when the definition of asthma exacerbation was tightened (from diurnal PEF variation over 10% to over 20%), suggesting short-term ozone exposure exhibited a stronger effect on severe exacerbation of asthma. Consistently, we found that PEF variation significantly increased with ozone exposure. Similarly, Zheng et al. reported a relatively higher risk for asthma hospitalization (relative risk = 1.014) than for asthma emergency room visits (relative risk = 1.006), where emergency room visits for asthma are more common and often less severe than hospital admissions (9).

Notably, almost all these previous studies used asthma emergency room visits or hospitalizations as the study endpoints. The use of emergency room visits or hospitalizations as indicators of asthma exacerbations may introduce health outcome misclassification because some asthma patients may delay visiting or not visit the hospital for personal reasons, or may not be timely admitted due to inadequate hospital beds. In addition, using asthma emergency room visits or hospitalizations may ignore early asthma exacerbation or mild asthma exacerbation that may not have any noticeable symptoms. Different from these publications, our study used diurnal PEF variation to define asthma exacerbation, which could avoid the above-mentioned issues. Researchers also demonstrated that diurnal PEF variation is more responsive and more reliable than symptoms in denoting asthma exacerbations during the acute setting. Especially for patients who have reduced lung function but do not exhibit symptoms, regular PEF monitoring is preferred to identify asthma exacerbation (5). In our study, we did find that ozone exposure was related with an elevated risk of asthma exacerbation defined by diurnal PEF variation of more than 10%, suggesting that ozone may have an early adverse effect on the respiratory system in asthma patients.

Most environmental epidemiological studies have attempted to find possible environmental triggers of childhood asthma and ignored adult asthma. Study populations in our study ranged in age from 18 to 88 years. In subgroup analysis, we found that the effects of short-term ozone exposure on asthma exacerbation were more prominent in patients over 45 years old, which was consistent with previous epidemiological findings (29, 30). This may be attributed to the fact that older asthma patients are more vulnerable to acute exacerbations because their lung function is declining rapidly over time as their asthma progresses. In this study, we also observed that the effects were more pronounced in males than in females, which may be partly explained by the apparent difference in smoking status between males and females. Previous studies have found that excessive tobacco use may increase the risk of hospitalization for asthma by promoting an ozone-producing inflammatory response (31). In China, the prevalence of smoking is greatly higher in males than in females, which makes our findings somewhat reasonable. Moreover, in line with a meta-analysis (27), we found that ozone exposure showed relatively higher effects on asthma exacerbation in the warm season than in the cool season. Ambient ozone concentration is typically higher in the warm season, which may partially explain seasonal differences in the effects (32). Another possible reason is that people prefer to be outside during the warm season, which may increase individual ozone exposure (33).

The biological plausibility of our findings that the risk of asthma exacerbation may increase with higher levels of ozone exposure is widely supported by animal studies and human controlled-exposure trials (28, 34). As a strong oxidant, inhaled ozone can cause direct damage to airway epithelial cells, thereby triggering asthma through a series of chain reactions, such as promoting the release of “alarmins” and stimulating dendritic cells (35, 36). The human body has a certain antioxidant capacity to resist ozone-mediated cellular responses. However, when people are exposed to high concentrations of ozone or are persistently exposed to ozone, the antioxidant defenses may be overwhelmed and thereafter may cause an inflammatory response, which is an important mechanism in the acute exacerbation phase of asthma (37, 38). Moreover, in animal studies, short-term exposure to ozone has been shown to increase airway hyper responsiveness, another important feature of asthma attack (39). Previous studies also suggested that ozone exposure may contribute to asthma exacerbation by other mechanisms, such as neuroeffector mechanisms (40) and immune modifications (41).

This multi-city longitudinal study has several strengths. For example, we have a large dataset on lung function of adult asthma patients measured every morning and evening, which allows us to explore asthma exacerbation at the individual level with short-term ozone exposure. In addition, we used diurnal PEF variation to define asthma exacerbations, which has been shown to be a more sensitive indicator than emergency room visits or hospitalizations. The use of lung function to define acute asthma exacerbations enables us to recognize mild asthma in absence of obvious symptoms yet. Therefore, our study provides more reliable evidence for the relationship between ozone and asthma exacerbation compared with previous ecological studies (e.g., time-series studies).

However, our study still has some limitations. First, our study failed to distinguish between patients with a single asthmatic condition and those with asthma combined with other diseases (e.g., chronic obstructive pulmonary disease), who may have a different response to short-term ozone exposure (42). Second, we did not collect data on environmental allergens (e.g., pollen and dust mites) and asthma treatment or medication, thus we cannot completely rule out their potential impacts on asthma attacks. However, by intensive and dynamic lung function measurements and the use of the GAM model, the effects of residual confounding can be reduced. Third, we were unable to perform personal ozone monitoring because of large sample size and long duration of the study, which may introduce exposure measurement errors. However, previous studies have indicated that these measurement errors typically resulted in the underestimation of the risk and we believe that using ambient ozone concentration did not have substantial impacts on our conclusion (43, 44). Lastly, the Respiratory Home Platform has only been developed for asthma patients in selected Chinese cities at present, therefore one should be cautious when directly extending the findings to other populations.

## 5. Conclusion

Our results suggest that short-term ozone exposure can increase the risk of asthma exacerbations, even when the exacerbation was in the early stage, and also suggest that male and older asthma patients may be more sensitive to ozone air pollution. The effect of ozone on asthma exacerbation was more evident in the warm season. These findings will have important implications for the health management of asthma patients, especially for vulnerable populations in the warm season.

## Data availability statement

The datasets presented in this article are not readily available because individual data after deidentification only will be made available to researchers whose proposed use of the data has been approved by an independent review committee. Requests to access the datasets should be directed to YN, niuy@fudan.edu.cn.

## Ethics statement

The studies involving human participants were reviewed and approved by the Institutional Review Board in School of Public Health, Fudan University, reviewed and approved the study protocol (IRB#2021-04-0889). Written informed consent to participate in this study was provided by the participants' legal guardian/next of kin.

## Author contributions

XF: data curation, formal analysis, writing—original draft preparation, and visualization. SH: project administration, investigation, and data curation. YZ: methodology, investigation, and data curation. JL: methodology and data curation. YX: conceptualization and methodology. YN: conceptualization, writing—reviewing and editing, and funding acquisition. RC: conceptualization, supervision, and funding acquisition. All authors read and approved the final manuscript.
