# Supplementary material for: Short-term exposure to ozone and asthma exacerbation in adults: A longitudinal study in China
Source: Front Public Health. 2023 Jan 6;10:1070231. doi: 10.3389/fpubh.2022.1070231 (PMC9854395; doi:10.3389/fpubh.2022.1070231)
Supplement: Supplementary file 1 [file Data_Sheet_1.docx]

**Supplementary material**


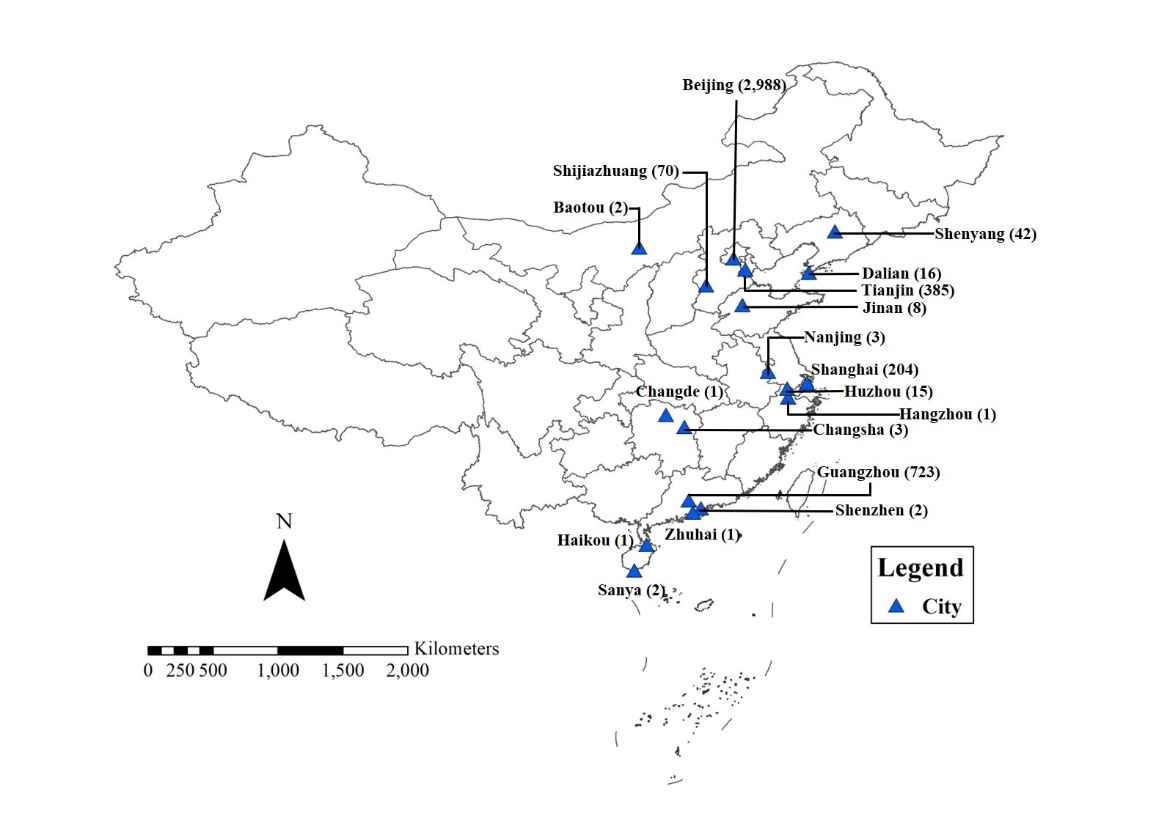


**Supplementary Figure 1.** Geographical distribution of 18 study cities. The number of participants in each city is in parentheses.

**Supplementary Table 1.** Descriptive statistics of air pollutants and meteorological conditions stratified by season and region in 18 Chinese cities, 2017–2020

| Variables | Mean | SD | Min | P_25_ | Median | P_75_ | Max |
| --- | --- | --- | --- | --- | --- | --- | --- |
| Air pollutants |  |  |  |  |  |  |  |
| O_3_, μg/m^3^ |  |  |  |  |  |  |  |
| Warm season | 115.5 | 57.3 | 4.5 | 72.5 | 107.6 | 155.8 | 264.5 |
| Cold season | 65.0 | 40.5 | 4.0 | 36.8 | 58.4 | 83.9 | 263.0 |
| Northern region | 88.2 | 57.1 | 4.0 | 47.6 | 73.1 | 120.2 | 264.5 |
| Southern region | 92.0 | 50.4 | 4.5 | 56.0 | 86.3 | 122.9 | 264.4 |
| PM_2.5_, μg/m^3^ |  |  |  |  |  |  |  |
| Warm season | 41.3 | 28.9 | 3.8 | 20.4 | 34.7 | 54.4 | 227.0 |
| Cold season | 52.3 | 45.2 | 3.8 | 20.4 | 37.8 | 68.9 | 246.1 |
| Northern region | 51.4 | 41.7 | 3.8 | 21.9 | 40.6 | 66.7 | 246.1 |
| Southern region | 34.5 | 23.7 | 4.0 | 18.8 | 28.0 | 43.2 | 227.0 |
| NO_2_, μg/m^3^ |  |  |  |  |  |  |  |
| Warm season | 43.0 | 20.3 | 7.5 | 29.0 | 38.8 | 52.7 | 127.9 |
| Cold season | 51.8 | 24.1 | 7.0 | 33.6 | 49.8 | 66.0 | 127.9 |
| Northern region | 44.9 | 21.0 | 7.0 | 29.4 | 41.5 | 57.0 | 127.5 |
| Southern region | 55.3 | 25.7 | 7.2 | 36.0 | 51.1 | 71.3 | 127.9 |
| SO_2_, μg/m^3^ |  |  |  |  |  |  |  |
| Warm season | 7.0 | 6.8 | 1.1 | 2.9 | 4.6 | 8.9 | 60.3 |
| Cold season | 10.8 | 9.2 | 1.2 | 4.6 | 8.2 | 13.8 | 61.3 |
| Northern region | 9.1 | 9.3 | 1.1 | 3.0 | 5.6 | 11.6 | 61.3 |
| Southern region | 8.7 | 4.8 | 1.2 | 5.0 | 7.9 | 11.0 | 58.9 |
| CO, mg/m^3^ |  |  |  |  |  |  |  |
| Warm season | 0.8 | 0.3 | 0.2 | 0.6 | 0.8 | 1.0 | 2.9 |
| Cold season | 0.9 | 0.5 | 0.2 | 0.6 | 0.9 | 1.2 | 2.9 |
| Northern region | 0.9 | 0.5 | 0.2 | 0.5 | 0.8 | 1.1 | 2.9 |
| Southern region | 0.8 | 0.3 | 0.2 | 0.6 | 0.8 | 1.0 | 2.3 |
| Meteorologic conditions |  |  |  |  |  |  |  |
| Temperature, °C |  |  |  |  |  |  |  |
| Warm season | 23.3 | 5.3 | 3.1 | 20.6 | 24.9 | 26.9 | 32.3 |
| Cold season | 5.8 | 8.1 | -13.5 | -0.9 | 4.3 | 12.3 | 27.0 |
| Northern region | 12.4 | 11.8 | -13.5 | 1.5 | 12.3 | 23.9 | 32.3 |
| Southern region | 19.1 | 7.3 | -6.0 | 14.2 | 20.5 | 25.5 | 31.0 |
| Relative humidity, % |  |  |  |  |  |  |  |
| Warm season | 66.7 | 20.6 | 14.4 | 52.4 | 68.5 | 84.6 | 98.0 |
| Cold season | 50.0 | 24.4 | 13.1 | 30.0 | 44.1 | 69.2 | 98.0 |
| Northern region | 48.6 | 19.6 | 13.1 | 32.2 | 48.0 | 64.6 | 97.9 |
| Southern region | 85.6 | 11.4 | 19.0 | 80.7 | 89.0 | 94.0 | 98.0 |

Abbreviations: SD: standard deviation; Min: minimum; Max: maximum; P_25_: the 25^th^ percentile; P_75_: the 75^th^ percentile; O_3_: ozone; PM_2.5_: particulate matter with an aerodynamic diameter < 2.5μm; SO_2_: sulfur dioxide; NO_2_: nitrogen dioxide; CO: carbon monoxide

**Supplementary Table 2.** Spearman correlation coefficients among air pollutants and meteorologic conditions in 18 Chinese cities, 2017–2020

|  | O_3_ | PM_2.5_ | SO_2_ | NO_2_ | CO | Temperature |
| --- | --- | --- | --- | --- | --- | --- |
| PM_2.5_ | 0.068* |  |  |  |  |  |
| SO_2_ | -0.094* | 0.420* |  |  |  |  |
| NO_2_ | -0.258* | 0.577* | 0.507* |  |  |  |
| CO | -0.144* | 0.744* | 0.495* | 0.649* |  |  |
| Temperature | 0.663* | -0.084* | -0.242* | -0.209* | -0.097* |  |
| Relative humidity | 0.086* | 0.135* | 0.029* | 0.146* | 0.296* | 0.512* |

* Statistically significant results (p < 0.01).

Abbreviations: O_3_: ozone; PM_2.5_: particulate matter with an aerodynamic diameter < 2.5μm; SO_2_: sulfur dioxide; NO_2_: nitrogen dioxide; CO: carbon monoxide.

**Supplementary Table 3.** Change in diurnal PEF variation associated with a 10μg/m^3^ increase in ozone concentrations, stratified by sex, age, season of lung function tests, and region

|  | Change (95% Confidence Interval) |
| --- | --- |
| Main model | 0.04% (0.02%, 0.06%) |
| Stratification |  |
| Sex |  |
| Male | 0.09% (0.06%, 0.13%) |
| Female | 0.00% (-0.03%, 0.03%) |
| Age |  |
| 45-88 | 0.06% (0.03%, 0.10%) |
| 18-44 | 0.01% (-0.02%, 0.04%) |
| Season ^a^ |  |
| Warm | 0.05% (0.02%, 0.08%) |
| Cool | 0.01% (-0.03%, 0.05%) |
| Region |  |
| South | 0.03% (-0.01%, 0.07%) |
| North | 0.05% (0.02%, 0.08%) |

Abbreviations: PEF: peak expiratory flow.

^a^: Warm season was defined as May to October; cool season was defined as November to April.
